# Palatal fibroblasts reduce osteoclastogenesis in murine bone marrow cultures

**DOI:** 10.1186/s12903-016-0195-y

**Published:** 2016-03-17

**Authors:** Victoria Voisin, Jordi Caballé-Serrano, Anton Sculean, Reinhard Gruber

**Affiliations:** Department of Periodontology, School of Dental Medicine, University of Bern, Bern, Switzerland; Laboratory of Oral Cell Biology, School of Dental Medicine, University of Bern, Bern, Switzerland; Department of Oral Surgery and Stomatology, School of Dental Medicine, University of Bern, Bern, Switzerland; Department of Oral and Maxillofacial Surgery, School of Dental Medicine, Universitat Internacional de Catalunya, Barcelona, Spain; Department of Preventive, Restorative and Pediatric Dentistry, School of Dental Medicine, University of Bern, Bern, Switzerland; Department of Oral Biology, Medical University of Vienna, Wien, Austria

**Keywords:** Palatal fibroblasts, Osteoclast, Macrophages, Co-culture, Inserts, Murine bone marrow cultures

## Abstract

**Background:**

Preclinical studies support the assumption that connective tissue grafts preserve the alveolar bone from resorption; the underlying cellular mechanisms, however, remain unknown. The cellular mechanisms may be attributed to the paracrine activity of the palatal fibroblasts. It was thus reasonable to suggest that palatal connective tissue grafts reduce the formation of osteoclasts.

**Methods:**

To test this hypothesis, human palatal fibroblasts were examined for their capacity to modulate the formation of osteoclasts in murine bone marrow cultures exposed to RANKL, M-CSF and TGF-β1. Osteoclastogenesis was determined by tartrate-resistant acid phosphatase (TRAP) staining and gene expression analysis. The formation of antigen presenting cells was based on the expression of CD14 and costimmulatory molecules of antigen presenting cells. The paracrine interaction of fibroblasts and the bone marrow was modeled in vitro with inserts of cell-occlusive membranes.

**Results:**

In cocultures without cell-to-cell contact, palatal fibroblasts caused a decrease in the expression of the osteoclast marker genes in bone marrow cells; calcitonin receptors, cathepsin K, TRAP, and osteoclast-associated receptor. Also the number of TRAP positive multinucleated cells was decreased in the presence of fibroblasts. Notably, palatal fibroblasts increased the expression of CD14 and the co-stimulatory proteins CD40, CD80, and CD86 in bone marrow cells. Bone marrow cells had no considerable impact on fibroblast viability and proliferation marker genes. With regard to cell distribution, osteoclasts were most prominent in the center of the membranes, while fibroblasts accumulated immediately adjacent to the border of the insert forming a ring-like structure on the surface of the culture plate.

**Conclusion:**

The data suggest that palatal fibroblasts provide a paracrine environment that reduces osteoclastogenesis and increases markers of antigen presenting cells. Morover, the paracrine model revealed a joint activity between palatal fibroblasts and bone marrow cells visualized by the characteristic cell distribution in the two separated compartments.

## Background

Palatal connective tissue grafts are used in periodontology for root coverage and to treat gingival recession defects [1]. Palatal connective tissue grafts have also been proposed to improve esthetic parameters when buccal bone is thin or resorbed in implant dentistry and prosthetics [2–6]. Depending on the technique used, palatal connective tissue grafts can be placed in direct contact with alveolar bone. The question arises about the possible impact of the palatal connective tissue grafts on the alveolar bone. There is at least weak support for a potential role of connective tissue grafts in preserving the underlying bone. Connective tissue graft placed at the buccal aspect of the bony wall, immediately after tooth extraction, yielded a reasonable preservation of the hard tissues [7]. Moreover, gingival connective tissue grafts can reduce the resorption of the marginal crest in canine models [8]. Taken together, both preclinical studies support the assumption that connective tissue grafts can preserve the alveolar bone; the underlying cellular mechanism however remains unknown.

The cellular mechanism may be attributed to the paracrine activity of the palatal fibroblasts. This assumption is supported by in vitro experiments with isolated gingiva fibroblasts and their ability to influence osteoclastogenesis, the formation of bone-resorbing cells. For example, conditioned medium obtained from gingiva fibroblasts and periodontal ligament fibroblasts inhibit the formation of osteoclast-like cells in mouse bone marrow cultures [9–11]. It is thus likely that paracrine factors released from palatal fibroblasts can reduce the process of osteoclastogenesis. More intriguing is to understand the molecular mechanism how the paracrine factors released from palatal fibroblasts change the expression of genes representing osteoclasts, but also the antigen presenting cells, both originating fom hematopoetic precursor cells.

Osteoclastogenesis in murine bone marrow cultures requires the presences of receptor activator of nuclear factor kappa-B ligand (RANKL), M-CSF, and the respective receptors [12]. Osteoclasts typically are multinucleated and express tartrate-resistant acid phosphatase (TRAP), cathepsin k (CatK) and the calcitonin receptor (CTR). Osteoclasts also express co-stimulatory molecules activating the immunoreceptor tyrosine-based activation motif (ITAM)-dependent pathway [13]. Osteoclast-associated receptor (OSCAR) and triggering receptor expressed in myeloid cells (TREM2) are receptors that are associated with the respective adaptor molecules Fc receptor common gamma chain (FcRγ) and DNAX-activating protein 12 kDa (DAP12), respectively [13]. When the differentiation shifts towards a macrophage phenotype, the expression of CD14, a co-receptor for lipopolysaccharide signaling, and the co-stimulatory proteins CD40, CD80 and CD86, commonly expressed in antigen presenting cells such as monocytes and macrophages, rise [14, 15].

Aim of this present pilot study was to investigate the impact of the paracrine environment of palatal fibroblasts on osteoclastogenesis from bone marrow precursors using a in vitro model where both cell sources are separated by cell occlusive membranes.

## Methods

### Human palatal fibroblast

Human palatal fibroblasts were prepared from explant cultures of three independent donors after approval of the ethical committee of the Medical University of Vienna (EK Nr. 631/2007). Fibroblasts that grew out from the explants and had not undergone more than five passages were used. Fibroblasts were cultivated in Dubeccos Modified Eagle Medium (DMEM) with antibiotics (Invitrogen Corporation, Carlsbad, CA, USA)) and plated at 100,000 cells/cm^2^ in the respective 12-well plates (Greiner-Bio-One GmbH). After the cultivation period, staining of the fibroblast was performed with DiffQuik (Roche Diagnostic Systems, Basel, Switzerland).

### Murine bone marrow cultures

Bone marrow cells were prepared by flushing femur and tibiae of 4 to 8 weeks old female Balb/c mice (BE76/12 Veterinärdienst des Kantons Bern) and seeded into ThinCert™ cell culture inserts (12 well format with nominal pore sizes of 0.4, μm; Greiner-Bio-One GmbH, Frickenhausen, Germany) at one million cells per cm^2^ in Eagle’s Minimum Essential Medium-Alpha Modification (αMEM) supplemented with 10 % fetal calf serum (FCS), antibiotics, M-CSF at 25 μg/ml, TGF-β1 at 10 μg/ml, and RANKL at 25 μg/ml. Bone marrow cultures were then transferred to the plates that contained the fibroblasts. Recombinant proteins were purchased from Prospec (Ness-Ziona, Israel). Bone marrow cells were cultivated with and without the fibroblast. Both cell types remain separated by the cell occlusive membrane of the ThinCert™ cell culture inserts. Staining for TRAP (Sigma Aldrich, St. Louis, MO) was performed at day five. Cells were considered osteoclasts when positive for TRAP and having three or more nuclei as observed by light microscopy.

### Expression of marker genes in murine bone marrow cultures

RNA was isolated from the bone marrow cultures and the palatal fibroblasts using the High Pure RNA Isolation Kit (Roche Applied Science, Rotkreuz, Switzerland). Reverse transcription (RT) was performed with Transcriptor Universal cDNA Master, and PCR analyses were done in triplicates using TaqMan Universal PCR Master Mix (Applied Biosystems, Carlsbad, CA) or the FastStart Universal Probe Master Rox on a 7500 Real-Time PCR System (Roche). Bone marrow cells were analyzed for the following genes: SybrGreen: RANK, C-FMS, TRAF6, C-FOS, MITF, PU1, NFATC-1, DC-STAMP, ATP6V0D2, CD14, CD40, CD80, CD86, CD11C; TaqMan (Applied Biosystems, Carlsbad, CA): CTR, CATK, TRAP, OSCAR, TREM2, DAP12, FCRγ. Fibroblasts were analyzed for the following genes: BCL2, PLK1, CCNB1, CCNE1, CCND1, MYBL2, GAPDH, BAD, BAX, and BCL-XL. Data were normalized for β-actin using the ΔΔCt method (Tables 1 and 2).

**Table 1:** SybrGreen Primers for RT-PCR

Gene	Forward primer	Reverse primer
m 18 s	tccagcacattttgcgagta	cagtgatggcgaaggctatt
m βactin	ctaaggccaaccgtgaaaag	accagaggcatacagggaca
m RANK	gtgctgctcgttccactg	agatgctcataatgcctctcct
m c-fms	gaccatggtgaatggtaggg	ggataacgttgaatcccactg
m TRAF6	ttgcacattcagtgtttttgg	tgcaagtgtcgtgccaag
m c-fos	gcaactttctatgacactgaaacac	tctctctagggctgcattgg
m MITF	gacaccagccataaacgtca	ttttccaggtgggtctgc
m PU1	ggagaagctgatggcttgg	caggcgaatctttttcttgc
m NFATc-1	ccgttgcttccagaaaataaca	tgtgggatgtgaactcggaa
m DC-Stamp	aagctccttgagaaacgatca	caggactggaaaccagaaatg
m Atp6vOd2	aagcctttgtttgacgctgt	gccagcacattcatctgtacc
m CD14	aaagaaactgaagcctttctcg	agcaacaagccaagcacac
m CD40	gagtcagactaatgtcatctgtggtt	accccgaaaatggtgatg
m CD80	tcgtctttcacaagtgtcttcag	ttgccagtagattcggtcttc
m CD86	gaagccgaatcagcctagc	cagcgttactatcccgctct
m CD11c	gagccagaacttcccaactg	tcaggaacacgatgtcttgg
h βactin	ccaaccgcgagaagatga	ccagaggcgtacagggatag
h BCL2	caggagaatggataaggcaaa	ccagccagatttaggttcaaa
h PLK1	aaccgagttattcatcgagacc	ttggttgccagtccaaaatc
h CCNB1	cctccggtgttctgcttc	ttcagcattaattttcgagttcc
h CCNE1	ggccaaaatcgacaggac	gggtctgcacagactgcat
h CCND1	gccgagaagctgtgcatc	ccacttgagcttgttcacca
h MYBL2	gtcaaatggacccatgagga	gtcagtgcggttagggaagt
h GAPDH	agccacatcgctcagacac	gcccaatacgaccaaatc
h BAD	cgagtttgtggactcctttaaga	caccaggactggaagactcg
h BAX	agcaaactggtgctcaagg	tcttggatccagcccaac
h BCL XL	agccttggatccaggagaa	agcggttgaagcgttcct

**Table 2:** TaqMan Primers Assay ID for RT-PCR

m CTR	Mm 00432282_m1
m CatK	Mm 00484039_m1
m TRAP	Mm 00475698_m1
m Oscar	Mm 00558665_m1
m Trem2	Mm 04209424_g1
m Dap12	Mm 00449152_m1
m FcRγ	Mm 02343757_m1

### Statistical analysis

Cell cultures were done using at least tree different fibroblast donors. The two groups were compared with the paired-*T* test with α < 5 %.

## Results

### Palatal fibroblasts inhibit the osteoclast-like cells pathway

To determine the impact of palatal fibroblast on osteoclastogenesis, a paracrine co-culture model based on inserts was performed. A main finding was that the presence of palatal fibroblasts in the lower chamber decreased the expression of the typical osteoclast marker genes CTR, CATK, TRAP and OSCAR by around 30 % (*p <* 0.05), but not the other co-stimulatory molecules of osteoclastogenesis. RANK and c-fms also remained unchanged. Moreover, TRAP staining was visibly reduced in the presence of palatal fibroblast. Mean number of osteoclasts in co-culture with fibroblasts per region of interest, randomly selected in each well: 11.7 (SD 6.7). Mean number of osteoclasts without fibroblasts per region of interest was 53.0 (SD 12.8; *p <* 0.05). Importantly, the presence of palatal fibroblasts increased the expression of CD14 and the co-stimulatory proteins CD40, CD80 and CD86 by around 2-4-fold (*p <* 0.05) compared to the respective controls without the presence of palatal fibroblasts (Table 3). Thus, the data support the concept that the palatal fibroblasts favor the development of antigen presenting cells rather than an osteoclast phenotype. Bone marrow cells did not affect the viability or the proliferation of the palatal fibroblasts, as indicated by the expression analysis of apoptosis and cell cycle related genes (data not shown).

**Table 3:** RT-PCR of osteoclast-like cells (OCL) in co-culture with palate fibroblasts (PF) using M-CSF, RANKL and TGF- β1 compared to co-cultures without PF, with mean values and standard deviation

Target genes	CD14	CD40	CD80	CD86	CTR	CatK	TRAP	OSCAR
Mean	3.13	2.48	1.57	4.31	0.63	0.71	0.75	0.62
Standard Deviation	0.66	0.82	0.53	1.42	0.31	0.34	0.31	0.30

The genes were cluster of differentiation 14, 40, 80 and 86 (CD), calcitonin receptor (CTR), cathepsin K (CatK), tartrate-Resistant Acid Phosphatase 5 (TRAP), osteoclast-associated immunoglobulin-like receptor (OSCAR). The table represents the mean and standard deviation of *n =* 6 resulting from two independent experiments with fibroblasts from three different donors. All mean values were different compared to controls without fibroblasts (*p <* 0.05)

### Characteristic distribution of osteoclasts and palatal fibroblasts in the insert cultures

**Fig. 1:**
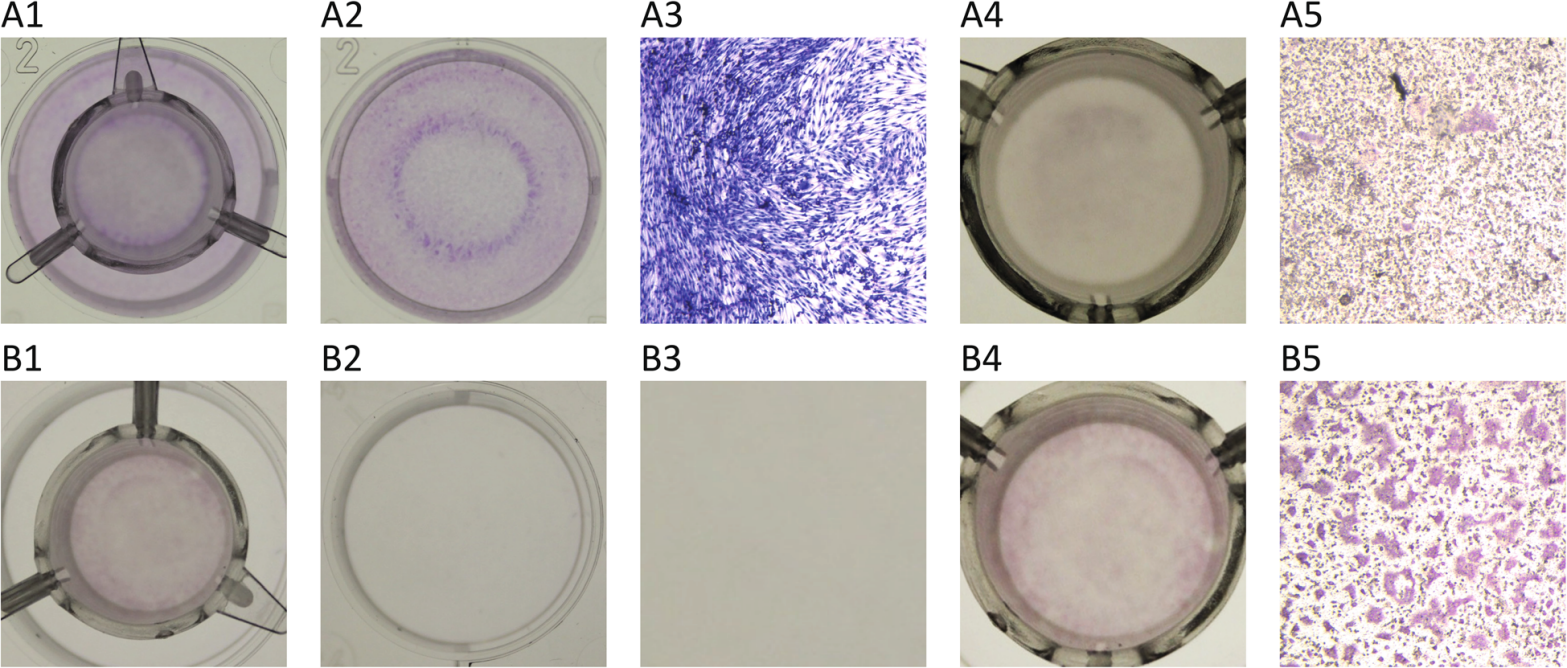
Staining of fibroblasts in wells and osteoclast-like cells in inserts after incubating murine bone marrow cells for 5 days with M-CSF, RANKL and TGF-β1 (MRT). Combined cultures with fibroblasts and osteoclasts (**a1**) showed an accumulation of the fibroblasts immediately adjacent to the border of the insert, forming a ring of fibroblasts at a high cell density (**a2**, **a3**). Moreover, palatal fibroblast had a major impact on the distribution of the osteoclasts: osteoclasts were prominent in the center of the insert (**a4**, **a5**). In cultures were no fibroblasts were present (**b1**, **b2**, **b3**), the TRAP-staining of the osteoclast-like cells showed a reduction in number and were more uniformly distributed compared to osteoclast-cells that had interaction with fibroblasts (**b4**, **b5**)

Palatal fibroblast had a major impact on the distribution of the osteoclasts. TRAP positive osteoclasts were prominent in the center of the insert (Fig. 1). Another phenomenon occurred towards the distribution of the palatal fibroblasts. The fibroblasts accumulated immediately adjacent to the border of the insert, forming a ring of high cell density (Fig. 1). These observations basically support that palatal fibroblasts reduce the formation of osteoclasts in a paracrine mode of action, but also that the bone marrow cells can attract fibroblasts in vitro. Thus, the paracrine model based on cell occlusive membranes with bone marrow cells in the inserts and fibroblasts in the lower compartment of the culture plate revealed a mutual activity visualized by the differential cell distribution.

## Discussion

The main finding of the present study was that palatal fibroblasts created a paracrine environment that moderately suppressed osteoclastogenesis while shifting the differentiation towards a macrophage phenotype. Osteoclast master genes were downregulated in the presence of fibroblasts, while genes characteristic for antigen presenting cells were upregulated. RANK and c-fms remained unchanged suggesting that the differentiation shift can not be explained by a decrease in responsiveness to the respective ligands, with M-CSF being critical also for monocyte development. Moreover, we observed a characteristic in vitro cell distribution, with osteoclasts remaining in the centers of the inserts, while the fibroblasts accumulated in a ring-like structure.

These in vitro findings are interesting because they basically support the observations from preclinical studies that connective tissue grafts reduce the resorption of the buccal bony wall [7, 8]. It is not surprising that reduced osteoclastogenesis goes along with a shift toward antigen presenting cells. Similar observations were made with saliva [15], and also doxycycline and minocycline induced the expression of dendritic cell markers, CD11c and CD86, in bone marrow cells in the presence of RANKL [14]. Taken together, the data provide insights into a possible paracrine mechanism on how palatal fibroblasts can reduce osteoclastogenesis while simultaneously supporting innate immunity indicated by expression of markers of antigen presenting cells, at least in vitro. Clearly this is not a conclusion, rather a suggestion supported be our preliminary data. Thus, the in vitro data and particularily the possible clinical relevance need to be interpreted carefully.

The pilot study has further limitations. This is an observational study showing a phenomenon, while the underlying molecular mechanisms remain unclear. The factors released by the palatal fibroblasts that are responsible for the observed shift from osteoclastogenesis towards the formation of as yet not further defined antigen presenting cells remains to be determined. One likely candidate is osteoprotegerin, a decoy-receptor for RANKL [12]. Osteoprotegerin is produced by fibroblasts, but to which extend and, if at all, this factor has caused the inhibition of osteoclastogenesis is yet unclear. Moreover, we used a xenogeneic system, which might have affected the overall outcome; human palatal fibroblasts and mouse bone marrow cells. However, molecules playing a role in osteoclast differentiation are evolutionary conserved and thus work inter-species [16]. Therefore, the use of mouse bone marrow cells in combination with human fibroblasts is appropriate as a proof-of-principles experiment that can be refined by the future use of interspecies in vitro models.

## Conclusions

Taken together, the present pilot study is a scientific basis for future research aiming to reveal the impact of the paracrine environment of palatal fibroblasts on osteoclastogenesis and the respective impact on the innate immune system. The present study is also a primer to further study the dynamics of cell movement created by the mutual paracrine environment.

### Availability of Data and Materials

The datasets supporting the conclusions of this article are included within the article.
